# Competition between Replicative and Translesion Polymerases during Homologous Recombination Repair in Drosophila

**DOI:** 10.1371/journal.pgen.1002659

**Published:** 2012-04-19

**Authors:** Daniel P. Kane, Michael Shusterman, Yikang Rong, Mitch McVey

**Affiliations:** 1 Department of Biology, Tufts University, Medford, Massachusetts, United States of America; 2 National Cancer Institute, Bethesda, Maryland, United States of America; 3 Program in Genetics, Tufts Sackler School of Graduate Biomedical Sciences, Boston, Massachusetts, United States of America; Stowers Institute for Medical Research, United States of America

## Abstract

In metazoans, the mechanism by which DNA is synthesized during homologous recombination repair of double-strand breaks is poorly understood. Specifically, the identities of the polymerase(s) that carry out repair synthesis and how they are recruited to repair sites are unclear. Here, we have investigated the roles of several different polymerases during homologous recombination repair in *Drosophila melanogaster*. Using a gap repair assay, we found that homologous recombination is impaired in Drosophila lacking DNA polymerase zeta and, to a lesser extent, polymerase eta. In addition, the Pol32 protein, part of the polymerase delta complex, is needed for repair requiring extensive synthesis. Loss of Rev1, which interacts with multiple translesion polymerases, results in increased synthesis during gap repair. Together, our findings support a model in which translesion polymerases and the polymerase delta complex compete during homologous recombination repair. In addition, they establish Rev1 as a crucial factor that regulates the extent of repair synthesis.

## Author Summary

DNA polymerases are required during both DNA replication and various types of DNA repair. DNA double-strand breaks are frequently repaired by homologous recombination, a conservative process in which DNA is copied into the break site from a similar template. The specific polymerases that operate during homologous recombination repair of DNA double-strand breaks have not been fully characterized in multicellular organisms. In this study, we created mutant strains of Drosophila lacking one or more DNA polymerases and determined their ability to synthesize large amounts of DNA during homologous recombination. We found that the error-prone translesion polymerases eta and zeta play overlapping roles during the initiation of synthesis, while the Pol32 subunit of the replicative polymerase delta complex is required for repair involving large amounts of synthesis. In addition, we showed that flies lacking the Rev1 translesion polymerase synthesize more DNA during gap repair than their normal counterparts. Our results demonstrate that both replicative and translesion polymerases are involved in homologous recombination and identify Rev1 as a protein that may regulate the access of various polymerases to double-strand break repair intermediates.

## Introduction

DNA double-strand breaks (DSBs) pose a serious threat to cell viability and genome integrity. DSBs can be repaired either by non-homologous end joining, in which the DSB ends are processed and directly ligated, potentially leading to loss of information and mutagenesis (reviewed in [1]), or by a group of repair mechanisms collectively known as homologous recombination (HR). During HR, DNA sequence that is lost due to the original damage event or during subsequent processing is recovered through invasion of a nearby template and copying of this sequence into the break site. Because HR makes use of an intact, homologous template, it is generally considered to be a conservative process. However, several studies have shown that HR repair can also be mutagenic, resulting in an increased mutation frequency both at the original break site [2] and at nearby sequences [3].

The initial events of HR involve the creation of single-stranded 3′ DNA ends, which are then coated with the Rad51 protein to form a nucleoprotein filament that conducts a genome-wide homology search (reviewed in [4]). Upon identification of a homologous template, a displacement loop (D-loop) is formed in which the duplex template is unwound and the invading broken strand pairs with its complement. This D-loop extends and/or migrates as repair synthesis continues. In one model of HR, termed synthesis-dependent strand annealing, the invading strand dissociates and anneals to single-stranded DNA on the broken duplex [5]. Single-stranded gaps are then filled in and the broken ends are ligated to complete repair.

Two general types of polymerases are potentially available for DNA synthesis during HR repair. Replicative polymerases are highly processive and replicate the bulk of DNA during S phase (reviewed in [6]). In contrast, translesion synthesis (TLS) polymerases are specialized for replication of damaged or abnormal templates (reviewed in [7], [8], [9]). Previous studies have provided conflicting results with regard to whether replicative or translesion DNA polymerases are predominantly used during HR repair synthesis.

In the budding yeast *Saccharomyces cerevisiae*, the catalytic subunits of the replicative polymerases (pol) delta and epsilon play important roles in repair synthesis during HR [2], [10], [11], [12]. Recently, purified pol delta from budding yeast was shown to efficiently extend D-loops in the presence of the polymerase clamp PCNA [13], confirming the *in vivo* findings. In addition, a non-essential subunit of pol delta, Pol32, is required for break-induced replication, a form of HR that requires extensive DNA synthesis [14].

TLS polymerases have also been implicated in HR repair. In chicken DT40 B lymphocytes, the absence of polymerases eta and zeta results in reduced gene conversion during antibody diversification and increased chromosomal abnormalities, respectively [15], [16]. Furthermore, *in vitro* studies using purified human proteins have identified a potential function for polymerase eta in extending D-loop intermediates [17], [18]. In budding yeast, TLS polymerases are not required for HR repair but localize at regions near DSBs [19] and contribute to mutagenesis near sites of DSBs [3], [20].

Thus, evidence from a variety of systems suggests that both replicative and error-prone TLS polymerases may be utilized during DSB repair. However, the roles of specific polymerases used during HR and how they are coordinated remains poorly defined. In this study, we present evidence that multiple TLS polymerases can function during the initial synthesis stage of HR repair and that they compete with polymerase delta during repair of a double-strand gap in Drosophila. Furthermore, we show that Rev1 may act to coordinate the initial recruitment of TLS polymerases, thereby preventing replicative polymerases from acting during early repair synthesis.

## Results

### Pol32 promotes extensive DNA synthesis during HR repair

**Figure 1:**
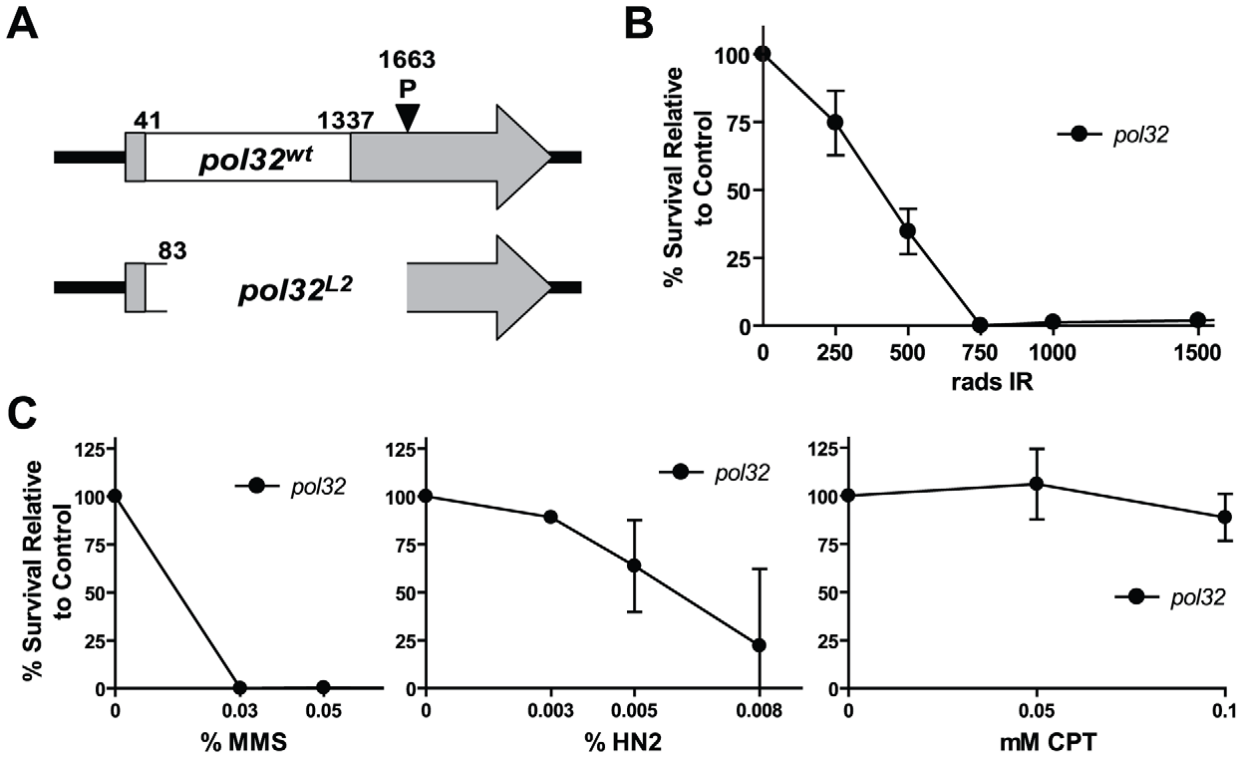
*pol32* mutants are sensitive to multiple DNA damaging agents. (A) A null allele (*L2*) of *POL32* (*CG3975*) was created through imprecise excision of a *P* element (EY15283) located in the 3′ untranslated region (UTR) of the *POL32* gene. White box indicates the *POL32* open reading frame; shaded regions, the UTRs; numbers indicate nucleotide position from start of transcription. (B) *pol32* mutants are sensitive to ionizing radiation (IR). Percent survival was calculated as the percentage of homozygote eclosion relative to an untreated control. (C) *pol32* mutants are sensitive to methyl methanesulfanate (MMS) and nitrogen mustard (HN2), but not camptothecin (CPT). Error bars represent the standard deviation for at least three trials.

We began by testing whether DNA polymerase delta is involved in HR. Currently, no fly stocks with viable mutations in the essential subunits of DNA polymerase delta exist. A putative Drosophila ortholog of Pol32, encoded by *CG3975*, has been previously identified. Drosophila Pol32 possesses conserved PCNA and polymerase alpha interacting motifs (Figure S1) [21]. We created multiple *CG3975* deletion alleles via imprecise excision of a *P* element located in the 3′ untranslated region of *CG3975* and performed a rigorous characterization of a potential null allele, *L2*, which eliminates almost the entire open reading frame (Figure 1A).

We exposed *CG3975^L2^* mutant larvae to increasing concentrations of various DNA damaging agents, and quantified the ability of these larvae to survive to adulthood, relative to untreated controls. The mutants were extremely sensitive to methyl methanesulfonate (MMS), nitrogen mustard, and ionizing radiation and mildly sensitive to hydroxyurea, but were not sensitive to camptothecin (Figure 1B, 1C and data not shown). The MMS sensitivity resembles that observed in *pol32* mutant yeast [22]. In addition, *CG3975^L2^* mutants are unable to replicate their DNA during early embryogenesis and are female sterile (Y. Rong, data not shown). Together, the conserved domain structure, mutagen sensitivity, and female sterility suggest that *CG3975* is a functional ortholog of Pol32. Thus, we will hereafter refer to *CG3975* as Pol32, acknowledging that additional studies are needed to confirm this assertion.

Previously, we have shown that *spn-A* mutants, which lack the Rad51 protein and are therefore unable to carry out the initial strand invasion steps of HR, are unable to survive ionizing radiation (IR) doses in excess of 750 rads [23]. Interestingly, *spn-A* and *pol32* mutants show similar survival defects following IR exposure (Figure 1B), suggesting that Pol32 might play a critical role in HR repair.

**Figure 2:**
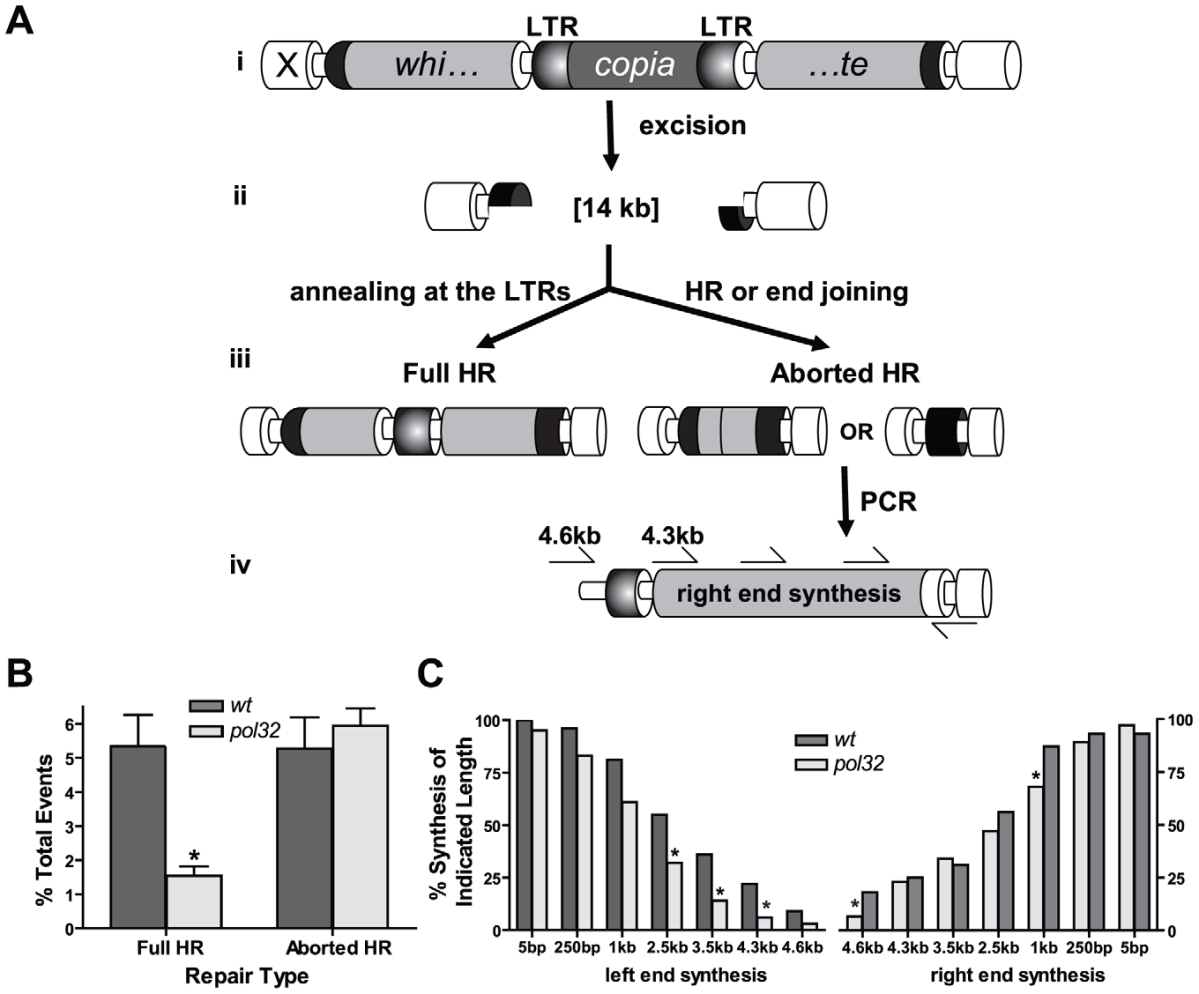
*pol32* mutants are impaired in DNA synthesis during HR repair. (A) The *P{w^a^}* site-specific repair assay. Expression of transposase in males possessing *P{w^a^}* (i) results in a 14 kilobase gap (ii) relative to an uncut sister chromatid. Full HR requires synthesis of the *white* gene and *copia* long terminal repeats (LTRs), followed by annealing at the LTRs (iii). Aborted HR results when end-joining repair occurs prior to synthesis of the entire *white* gene. Amount of repair synthesis in aborted HR repair events can be estimated by PCR (iv). (B) *pol32* mutants are significantly impaired in full HR repair relative to wildtype. Wildtype n = 55; *pol32* n = 120. Error bars represent standard errors. *P<0.05, Mann-Whitney test. (C) Repair synthesis is decreased in *pol32* mutants. Each bar represents the percentage of events with at least the indicated amount of synthesis. Right end: wildtype n = 55; *pol32* n = 151. Left end: wildtype n = 55 *pol32* n = 66. *P<0.05, Fisher's exact test.

To further characterize the role of Pol32 in HR repair, we utilized a site-specific DSB repair assay in which the mechanism of repair can be inferred using an eye color reporter construct [24]. We chose this assay because it imposes a demand for large amounts of repair synthesis and should therefore be extremely sensitive to genetic changes that alter polymerase activity. In the assay, dual DSBs are created on the same chromosome via excision of an *X* chromosome-linked *P{w^a^}* element, generating a 14 kb gap (Figure 2A). The *P{w^a^}* element contains a *white* gene driven by an Hsp70 promoter. Expression of *white* is decreased due to a *copia* retrotransposon insertion into an intron of *white*; females homozygous for the insertion have an apricot eye color. Following excision of *P{w^a^}* in the male pre-meiotic germline, repair usually initiates through HR, utilizing an unbroken sister chromatid as a template [24]. Repair products in males are recovered in female progeny that also inherit an intact *P{w^a^}* element from their mothers, and the frequency of three different types of repair events can be quantified using eye color as a reporter for the type of repair: (1) No excision of the *P{w^a^}* element or restoration of the intact transposon results in the original apricot eye color; (2) Repair that involves extensive synthesis (at least 4.5 kilobases from both ends, 9 kb total) and annealing at the long terminal repeats of *copia* allows for full expression of the *white* gene and results in a red eye color (hereafter referred to as “full HR”); (3) Repair in which end joining occurs immediately upon excision, or HR in which synthesis aborts prematurely before fully copying the *white* gene results in yellow-eyed flies (“aborted HR”). In the third case, the amount of repair synthesis that occurred prior to end joining can be estimated by PCR. Previously, we have found that end-joining repair without synthesis is an extremely rare event in wild-type flies [24]. Failed repair events in which HR aborts but end joining is not completed will presumably be lost to apoptosis and not recovered.

Strikingly, the frequency of full HR repair decreased by approximately 70% in *pol32* mutants (Figure 2B). This could reflect a requirement for Pol32 in annealing at the long terminal repeats of *copia* or a role of Pol32 in primary HR synthesis. Analysis of repair synthesis tract lengths supports the latter interpretation. Repair synthesis from the aborted HR products was shorter in *pol32* mutants, particularly as measured from the left end of *P{w^a^}*. The point where Pol32 becomes crucial appears as early as 2.5 kilobases from the left end of the break (Figure 2C). Overall, these results suggest that Drosophila Pol32 is important for HR repair involving extensive DNA synthesis. As aborted HR occurs at different distances on the left and right ends, we cannot rule out the possibility that Pol32 is required both to enhance pol delta processivity and also to promote synthesis through difficult to replicate, sequence-specific regions.

### Polymerases eta and zeta function in HR repair

**Figure 3:**
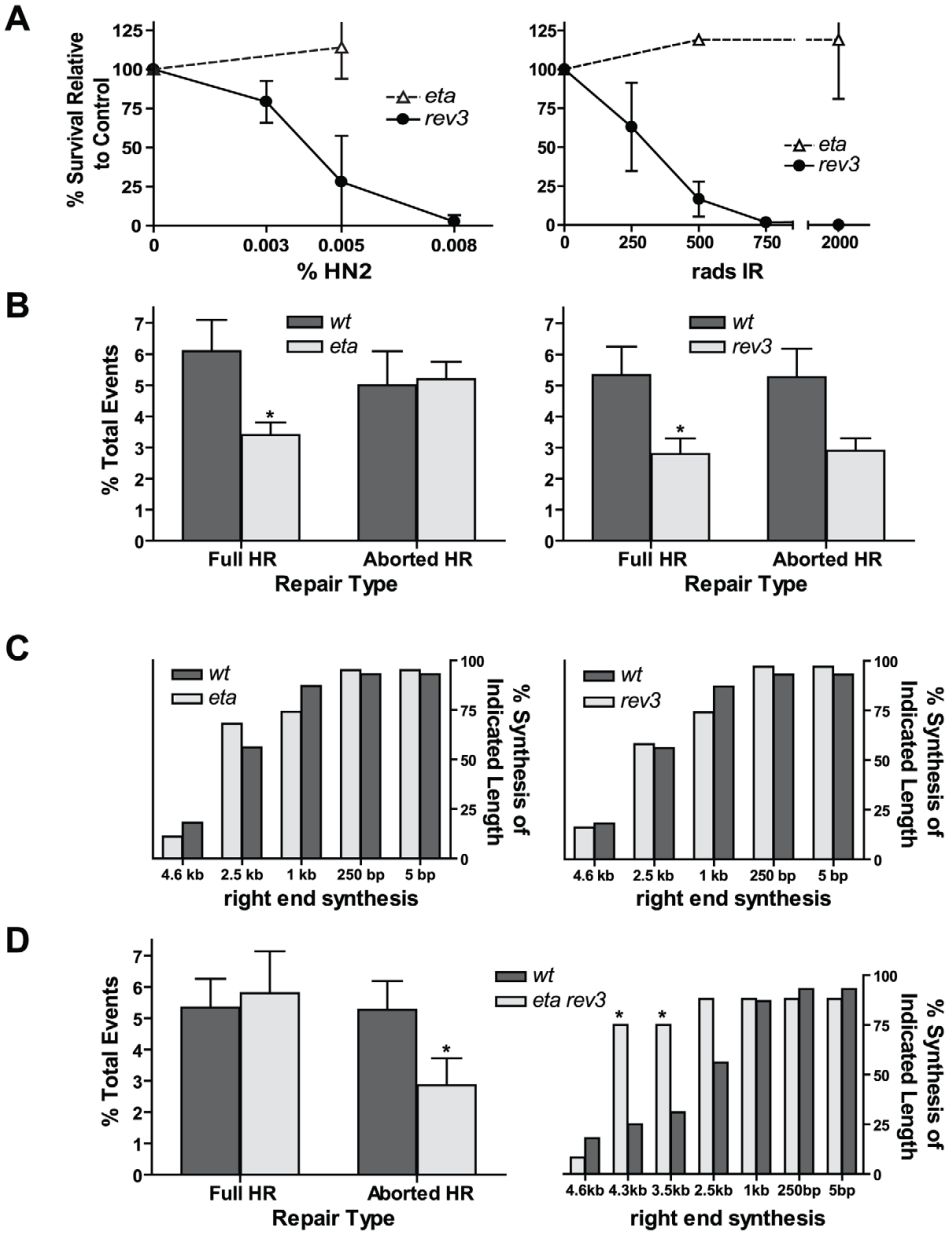
Flies lacking pol eta and the catalytic subunit of pol zeta are HR–deficient. (A) Flies lacking Rev3, but not pol eta, are sensitive to nitrogen mustard (HN2) and ionizing radiation (IR). (B) Both *pol eta* and *rev3* mutants have decreased full HR repair. Wildtype n = 43; *pol eta* n = 38; *rev3* n = 98. *P≤0.05, Mann-Whitney test. Wildtype versus *rev3* aborted HR repair, *P* = 0.062, Mann-Whitney test. (C) Repair synthesis is unchanged in the absence of pol eta (n = 19) or Rev3 (n = 38). (D) Left panel: *pol eta rev3* double mutants have no change in full HR, but a decrease in aborted HR (n = 85). *P<0.05, Mann-Whitney test. Right panel: repair synthesis tract lengths are increased in *pol eta rev3* mutants (n = 24). *P<0.05, Fisher's exact test.

We hypothesized that the residual repair synthesis that occurs in the absence of Pol32 could result from the action of either the core pol delta complex or from translesion polymerase activity. To test the latter possibility, we used imprecise *P* element excision to generate deletions in the coding regions of polymerase eta (encoded by *CG7143*) and Rev3 (the catalytic subunit of polymerase zeta, encoded by *mus205*) (Figure S2A). Larvae possessing each of these mutations were tested for their ability to survive exposure to various DNA damaging agents. Loss of pol eta resulted in severe sensitivity to ultraviolet (UV) radiation, but not to other mutagens (Figure 3A and Figure S2B). This likely reflects a need for pol eta to bypass UV-induced lesions [25], [26]. In contrast, *rev3* mutants were extremely sensitive to multiple mutagens, including ionizing radiation, MMS, and nitrogen mustard (Figure 3A and Figure S2B). As with the *pol32* mutants, the similar sensitivity of *rev3* and *spn-A* mutants to ionizing radiation suggests that polymerase zeta plays an important role in HR repair.

Next, we utilized the *P{w^a^}* assay to determine if flies lacking either pol eta or pol zeta were defective in HR repair of a site-specific DSB. Flies lacking pol eta had a 45% decrease in full HR repair relative to wildtype (Figure 3B, left), but the frequency of aborted HR was unchanged. Full HR repair in *rev3* mutants was also decreased relative to wildtype by 50% (Figure 3B, right). However, PCR analysis of aborted HR repair products revealed no significant difference in the synthesis tract lengths between repair events isolated from wildtype and *pol eta* or *rev3* mutants (Figure 3C). This was true for both the left and right ends of the repair products (data not shown). This indicates that DNA polymerases eta and zeta play a role in gap repair that is distinct from that of Pol32, which appears most important in repair contexts requiring multiple kilobases of synthesis. Because we observed no difference between repair tract lengths of aborted HR products for wildtype and *pol eta* or *rev3* mutants, the roles of these TLS polymerases may be limited to initiation of synthesis. Additionally, their roles may be partially redundant. From these data, we also could not rule out the possibility that the decrease in full HR events in *pol eta* and *rev3* mutants might be due to a defect in gap filling after dissociation and annealing (and not primary HR synthesis from the D-loop).

To determine if redundancy exists between TLS polymerases in HR synthesis, we constructed *pol eta rev3* double mutants. We initially predicted that since each single mutant showed a reduction in full HR events, the double mutant would display a further reduction in HR repair. Surprisingly, we observed no difference in the frequency of full HR repair for the *pol eta rev3* mutant compared to wildtype (Figure 3D, left). Additionally, repair tract lengths in aborted HR products from the double mutant were substantially increased compared to wildtype (Figure 3D, right). The increase in tract lengths suggests that pol eta and pol zeta act redundantly and, in their absence, repair synthesis is more extensive, increasing the chance of recovering full HR events relative to both single mutants. In addition, the change in synthesis tract lengths indicates that these two TLS polymerases act during primary HR synthesis and that their role is not limited to single-strand gap filling.

### Loss of Rev1 increases HR repair synthesis

Pol eta and pol zeta could function independently during HR synthesis, or they could be recruited to the site of the DSB by a common mechanism. In mice and flies, translesion polymerase Rev1 is known to interact with multiple translesion polymerases, including polymerases eta and zeta [27], [28], and these interactions are conserved in budding yeast [29]. Rev1 is highly upregulated in late S/G2 [30], which corresponds to the period of the cell cycle when HR is most active and when breaks induced by excision of the *P{w^a^}* element are being repaired. Rev1 has also been shown to be required to recruit polymerase zeta to sites of DSBs in yeast [19]. We therefore hypothesized that Rev1 might be acting to coordinate the recruitment of both pol eta and pol zeta to initial HR intermediates.

**Figure 4:**
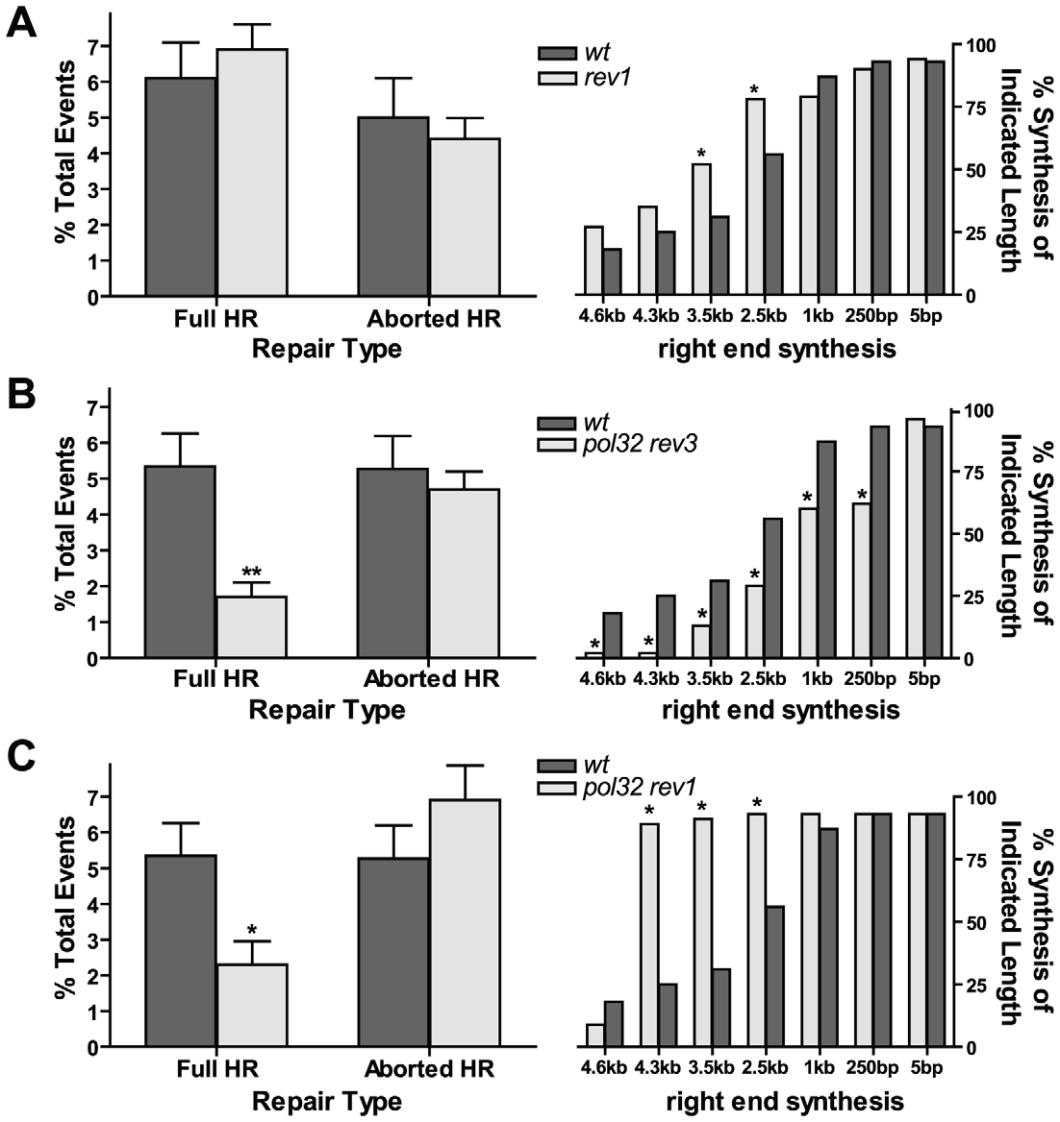
Rev1 regulates extent of repair synthesis during HR. (A) Left panel: HR efficiency is unchanged in *rev1* mutants (n = 104). Right panel: repair synthesis is increased in *rev1* mutants (n = 97). *P<0.05, Fisher's exact test. (B) Left panel: flies lacking both Pol32 and Rev3 have decreased full HR repair (n = 173). **P<0.01, Mann Whitney test. Right panel: aborted HR events have shorter synthesis tract lengths in *pol32 rev3* mutants (n = 45). (C) Left panel: flies lacking both Pol32 and Rev1 have decreased full HR repair (n = 34). *P<0.05, Mann-Whitney test. Right panel: aborted HR events from *pol32 rev1* mutants (n = 45) have increased synthesis tract lengths compared to wildtype.

To test this, we obtained a *rev1* mutant stock of flies with a *Minos* transposable element inserted into the *REV1* coding region (Figure S3A). We were unable to detect any *REV1* transcript by RT-PCR, suggesting that the transposon insertion is a null mutant (Figure S3B). The mutant also showed high sensitivity to ionizing radiation, indicative of a role for Rev1 in HR repair (Figure S3C). Interestingly, the *rev1* mutant phenotype in the *P{w^a^}* assay was qualitatively similar to that of the *pol eta rev3* double mutant: the percentage of full HR repair showed no difference relative to wildtype, while the repair synthesis tract lengths increased over that of wildtype (Figure 4A). However, the increase in tract lengths in the *rev1* mutants was not as high as that seen in *pol eta rev3* double mutants (Figure 3D; *P*<0.05 at 3.5 and 4.3 kb, Fisher's exact test). Thus, although repair synthesis is more processive in its absence, Rev1 does not appear to be absolutely required for the coordination of both pol eta and pol zeta during HR repair. Because *rev3* and *rev1* mutants are similarly sensitive to IR, we predict that the major role of Rev1 is to recruit pol zeta to early DSB repair intermediates.

### Translesion and replicative polymerases act competitively during HR

Our results indicate that both TLS and replicative polymerases are acting during HR repair of a double-strand gap. These polymerases could compete for D-loop substrates, with the amount of synthesis at any given point during HR dependent upon the processivity of the polymerase synthesizing at that moment. Alternatively, a mechanism for a coordinated polymerase switch may exist, where HR synthesis initiates with a TLS polymerase and later switches to a replicative polymerase. To explore these two possibilities, we created a *pol32 rev3* double mutant. Full HR repair was reduced 70%, similar to *pol32* single mutants, but repair synthesis tract lengths were reduced dramatically in aborted HR repair products compared to wildtype, with defects at distances as short as 250 base pairs and virtually no synthesis observed at distances ≥4.3 kilobases (Figure 4B). The synthesis defect was also more severe than that of the *pol32* single mutants (Figure 2C, right; *P*<0.05 at 0.25, 2.5, 3.5, and 4.3 kb, Fisher's exact test). This synergistic effect is consistent with the idea that pol delta (with Pol32) and pol zeta directly compete for HR intermediates. When both polymerases are impaired or eliminated, repair synthesis is greatly inhibited.

We reasoned that if the only function of Rev1 is to recruit polymerase zeta to sites of HR repair, then the phenotype of *pol32 rev3* and *pol32 rev1* mutants should be identical. To test this hypothesis, we performed the *P{w^a^}* assay in a *pol32 rev1* mutant background. Although full HR events were reduced by 60% in *pol32 rev1* mutants, repair synthesis tract lengths were increased dramatically over wildtype (Figure 4C). Thus, in the absence of both Pol32 and Rev1, initial synthesis appears to be more processive, but long-distance synthesis is reduced. These observations are consistent with data shown in Figure 2C and Figure 4A. In *rev1* mutants, repair synthesis is initially more processive, and in the absence of Pol32, repair synthesis is impaired at long distances. This combined phenotype is most pronounced when examining the repair tract lengths on the left end (Figure S4). However, because the *pol32 rev1* phenotype differs from that of the *pol32 rev3* mutant, this suggests that Rev1 might have two functions in gap repair: to recruit polymerase zeta and to exclude more processive polymerases from acting during the initial stages of repair synthesis.

## Discussion

**Figure 5:**
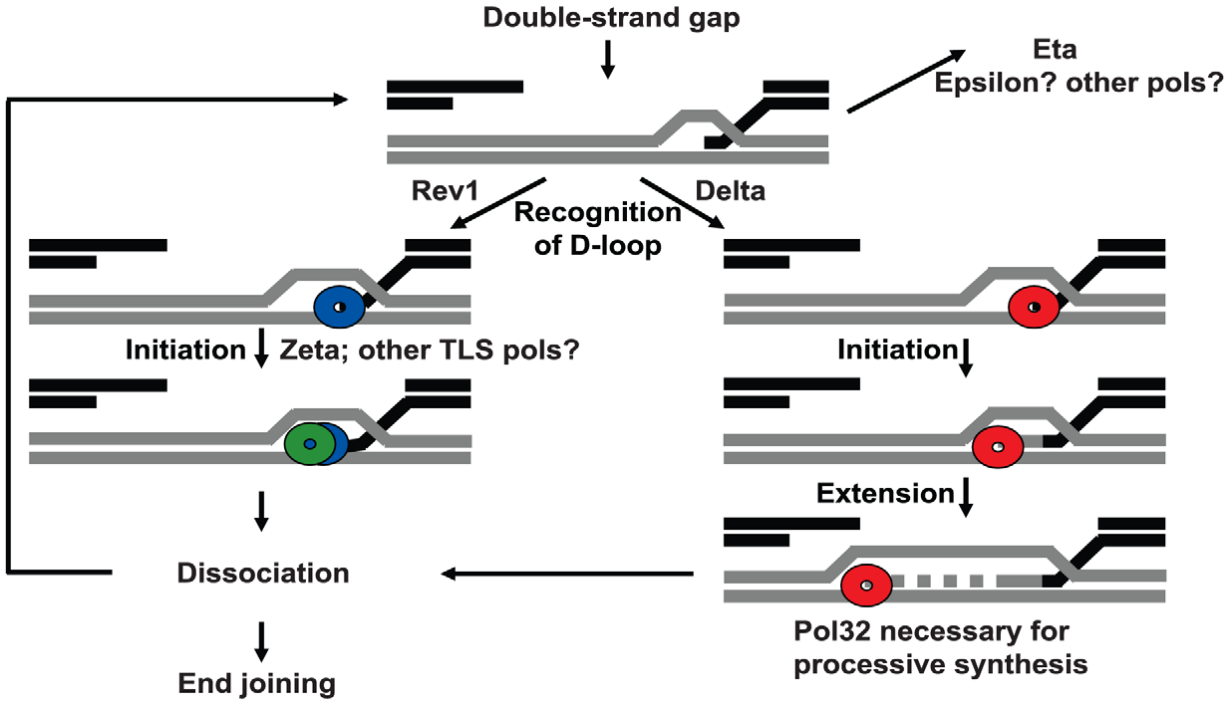
Model for polymerase action at a DSB. Multiple polymerases compete for access to D-loops. Following formation of a double-strand gap, Rev1 binds at the break site(s), recruits pol zeta, and blocks access of other polymerases. Initial synthesis is carried out by pol zeta, which readily dissociates. Repair can then be completed by end joining or another polymerase can bind and reinitiate synthesis. Binding of pol delta and its processivity subunit Pol32 to the D-loop results in processive synthesis and promotes repair of large gaps. Other polymerases, including pol eta, can act in backup roles. Elimination of Rev1 or multiple TLS polymerases increases the probability of pol delta recruitment leading to increased repair synthesis.

Taken together, our data suggest a model in which TLS polymerases and replicative polymerases compete for access to D-loop structures during initial HR repair synthesis (Figure 5). Based on the increased repair tract lengths that we observed in the absence of Rev1 (Figure 4A) and when both pol zeta and pol eta were missing (Figure 3D), we hypothesize that Rev1 and other translesion polymerases with low processivity are preferentially recruited to D-loops soon after they are formed. These polymerases may frequently dissociate, resulting in D-loop disassembly. Once the D-loop dissociates, reinvasion, polymerase binding, and extension can occur again, or repair can be completed by end joining [23], [31]. Increasing the frequency of dissociation may also increase the probability of failed repair and subsequent cell death.

In the absence of Rev1, a more processive polymerase (likely pol delta) can gain access to the D-loop intermediates, resulting in longer repair tract lengths. In cases when pol delta is loaded, Pol32 appears to be important for maintaining the processivity of the delta complex (Figure 2C). This is consistent with *in vitro* replication assays where Pol32 aids the processivity of pol delta in budding yeast [32] and *in vivo* assays where Pol32 is important for break-induced replication and gap repair [14], [33].

In yeast, Rev1 levels greatly increase during S/G2 [30]. If a similar upregulation occurs in Drosophila, this would increase the probability that Rev1 would arrive first at a DSB. Rev1 could directly bind to DSBs [34], or it could be recruited by an interaction between its BRCT domain and phosphoproteins that accumulate near the break site [19]. Drosophila Rev1 can interact with both pol eta and with Rev7, the non-catalytic subunit of pol zeta that forms a heterodimer with Rev3 [27]. Based on the phenotypes of the *rev3* and *pol eta* mutants (Figure 3A and 3B), we postulate that pol zeta is the primary TLS polymerase recruited by Rev1 at DSBs, but that pol eta can function in a backup capacity.

The preferential recruitment of non-processive TLS polymerases during HR initiation provides an explanation for previous findings that multiple strand invasions and rounds of synthesis occur during double-strand gap repair [23] and could also explain the template switching that occurs during the initial stages of break-induced replication [35]. Notably, the use of TLS polymerases as “first responders” might be particularly advantageous in instances where extensive synthesis might be unfavorable or energetically costly. As a corollary to this, large gaps that require extensive synthesis may be particularly difficult to repair by HR and may be ultimately repaired by end joining [36].

Two of the most significant findings from our study are: (1) multiple polymerases can initiate HR synthesis, and (2) the access of these polymerases to HR intermediates is likely regulated by Rev1. In support of the first conclusion, loss of both Pol32 and Rev3 results in extremely short synthesis tract lengths in aborted HR repair products (Figure 4B), suggesting that these polymerases act independently. Interestingly, a limited amount of repair synthesis is still observed in *pol32 rev3* mutants, suggesting that other polymerases are able to compensate to a certain degree in the absence of these subunits. The second conclusion arises from the difference in repair synthesis tract lengths between aborted HR repair products isolated from *pol32 rev3* (very short repair tracts, Figure 4B) and *pol32 rev1* (long repair tracts, Figure 4C) mutants. These results suggest that Rev1, even in the absence of pol zeta, can prevent access of processive, replicative polymerases (such as pol epsilon or the core pol delta complex) to HR intermediates. This idea is further supported by the decreased percentage of aborted HR repair events recovered from *rev3* mutants (Figure 3B and 3D). Perhaps in this mutant genotype, Rev1 also precludes repair by non-homologous end joining, resulting in cell death and a corresponding decrease in aborted HR repair.

Rev1 also interacts with Pol32 in budding yeast, and this binding prevents the interaction of Rev1 with pol zeta through Rev7 [29]. However, our data do not suggest that the Rev1-Pol32 interaction is being utilized to recruit the catalytic subunit of pol delta to sites of DSBs. If this were the case, repair synthesis tract lengths in *pol32 rev1* mutants should be equal to the *pol32* single mutant. Instead, repair tract lengths were increased in the double mutant (Figure 4A versus Figure 4C), supporting the idea of direct competition between TLS polymerases and pol delta, with Rev1 arriving first to either recruit pol zeta or to preclude pol delta. It has been shown that Rev1 can localize to sites of UV damage independently of pol zeta [37] and we postulate this can also occur at DSBs.

Our finding that significant redundancy exists between different polymerases in HR synthesis highlights an emerging theme in DNA repair. In many eukaryotes, precedent exists for the utilization of multiple DNA polymerases during various types of DNA repair. For example, in DT40 cells, mutants lacking polymerases eta, nu, and theta show reduced capacity for HR repair during immunoglobulin gene conversion [38]. In mammalian cells, polymerases delta, kappa, and epsilon all play active roles during nucleotide excision repair [39] and repair of interstrand crosslinks can involve a combination of six different translesion polymerases, depending on the type of crosslink and stage of the cell cycle (reviewed in [40]). Directly related to our findings, recent experiments with human cells demonstrate that knockdown of TLS polymerases zeta and Rev1 causes a >50% reduction in gene conversion following I-*Sce*I induction of a DSB [41]. Here, we have shown that, for a double-strand gap, TLS polymerases play a central role in the initiation of HR synthesis and directly compete with replicative polymerases. Future studies are needed to fully elucidate the mechanisms by which these different polymerases are recruited to sites of HR repair and to determine how polymerase choice is regulated.

## Materials and Methods

### Fly stocks and mutant creation

Flies were reared at 25°C on standard cornmeal agar medium. Stocks possessing *P* element and *Minos* insertions were obtained from Bloomington Stock Center or from the lab of Hugo Bellen. In some instances, *P* elements were crossed to a *Δ2–3* transposase source in a *mus309^N1^* mutant background to generate large deletion mutations, as described in [42]. The *mus309^N1^* mutation was removed before further experimentation.

### Mutagen sensitivity assays

For all tests, heterozygous mutants were mated in vials containing 5 mL of food and allowed to lay eggs for three days before being transferred to fresh vials for two additional days. One group of vials was treated with 250 µL of mutagen solution, while the other was treated with the same volume of vehicle control. For ionizing radiation studies, embryos were collected on grape-juice agar plates for 12 hours and allowed to develop to third instar larvae, then irradiated in a Gammator 1000 irradiator. For all other mutagens, progeny were treated as first instar larvae. Vehicle control was H_2_0 for all treatments except for camptothecin, in which DMSO in a 20% Tween, EtOH solution was used. Percent survival relative to control was calculated as the ratio of the percentage of homozygotes that eclosed in the treatment group relative to the expected number based on homozygote survival in the control group. Each experiment consisted of at least five independent vials, and error bars represent standard deviations of at least three independent replicates.

### Site-specific gap repair *P{w^a^}* assay

HR repair was monitored through the DSB created after excision of a *P{w^a^}* element as described previously ([43] and see text). A second chromosome transposase source *(CyO, H{w+,Δ2–3})* was used to excise *P{w^a^}* for *rev1* and *pol eta* single mutants, whereas all other experiments were performed with a third chromosome transposase source (*P{ry+, Δ2–3}*). Matched wildtype controls using the appropriate transposase source were done for each experiment (the same representative control for each respective transposase source is indicated throughout). Individual males possessing both *P{w^a^}* and the transposase source were mated to females homozygous for *P{w^a^}* and repair products were recovered in female progeny. Each vial was counted as an independent sample and statistical significance was calculated using the Mann-Whitney statistical test. Genomic DNA from flies possessing independent repair events was recovered [44] and PCR was carried out to estimate the extent of repair synthesis (see Text S1). Control tract lengths were obtained from excisions using the third chromosome transposase source.

## Supporting Information

Figure S1 ClustalW alignment of Pol32 in yeast, human and fly. Pol32 was originally identified in *Drosophila melanogaster* by Gray et al. (2004) based on its pol-alpha interacting domain (highlighted) and PCNA-interacting motif (underlined). Only the first 14 amino acids would be present in a theoretically expressed *pol32^L2^*.
(TIF)

Figure S2 Characterization of *pol eta* and *pol zeta* mutants. (A) Mutants of *pol eta* and *rev3* (*mus205*) were created through imprecise excision of a *P* element (*pol eta*: EY07711; *mus205*: EY20083). White box indicates open reading frame; shaded arrow, the untranslated regions; numbers indicate nucleotide position from start of transcription. (B) *pol eta* mutants are sensitive to ultraviolet radiation (UV) and *rev3* mutants are sensitive to methyl methanesulfonate (MMS). Percent survival was calculated as homozygote eclosion relative to an untreated control. Error bars represent the standard deviations of at least three trials.
(TIF)

Figure S3 Characterization of a *rev1* mutant. (A) Minos (Mi) transposable element insertion (MB11152) in the *REV1* coding region. (B) The Minos insertion is a null allele. RT-PCR was conducted using RNA isolated from MB11152 homozygous stocks. rp49 (ribosomal protein) was used as a control. Primers were designed to span an intron; PCR using genomic DNA (gDNA) produces a larger PCR product. MW = molecular weight marker. (C) Flies homozygous for the MB11152 insertion (*rev1* mutants) are sensitive to ionizing radiation (IR).
(TIF)

Figure S4 Left end repair synthesis in *pol32 rev1* mutants. Aborted HR events from *pol32 rev1* mutants (n = 45) have an intermediate phenotype relative to *pol32* and *rev1* single mutants. Each bar represents the percentage of events with at least the indicated amount of synthesis.
(TIF)

Text S1 Additional Methods. Expanded methods, including primers used, for the *P{w^a^}* assay.
(DOC)
