# Supplementary figures and images for: Competition between Replicative and Translesion Polymerases during Homologous Recombination Repair in Drosophila
Source: PLoS Genet. 2012 Apr 19;8(4):e1002659. doi: 10.1371/journal.pgen.1002659 (PMC3330096; doi:10.1371/journal.pgen.1002659)

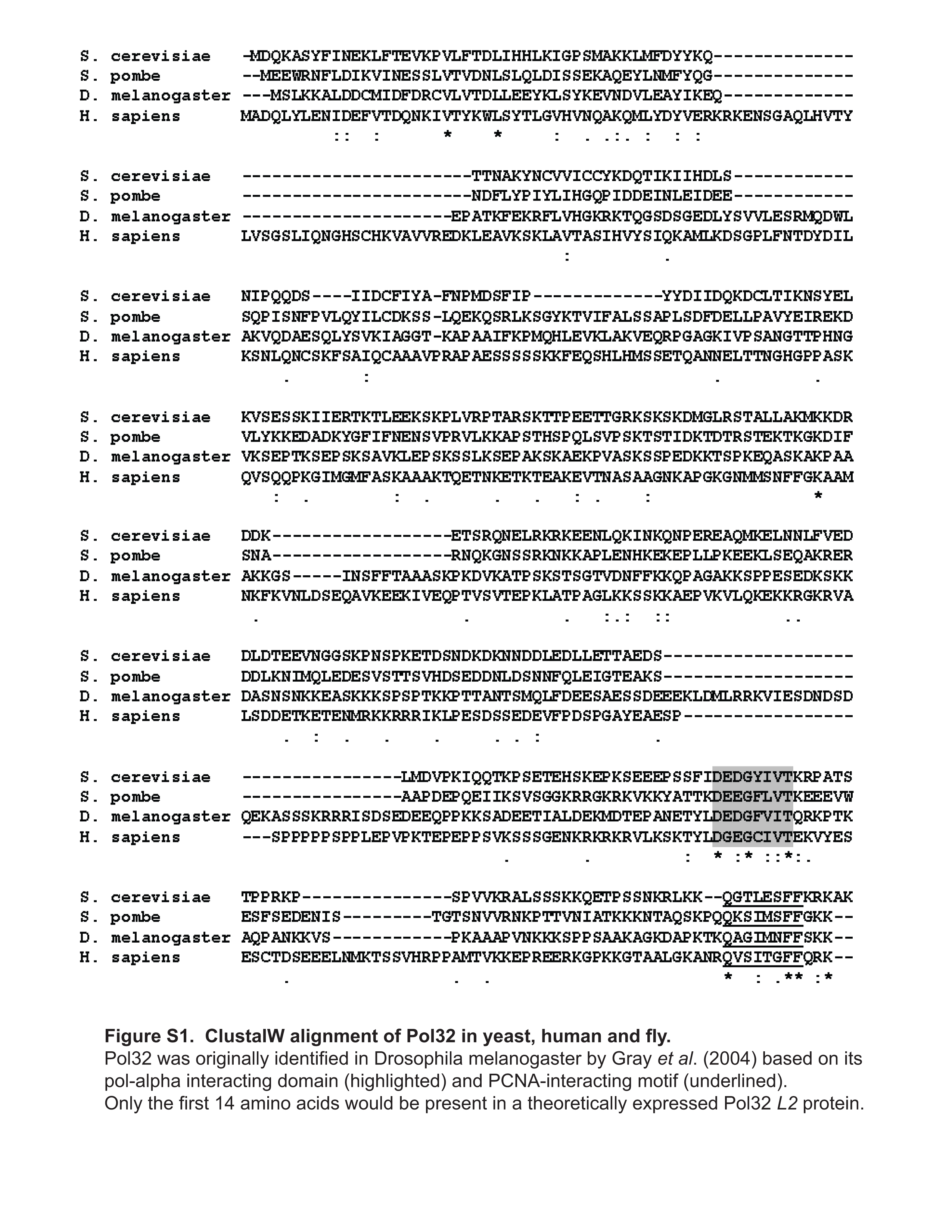

Supplement: Figure S1 — ClustalW alignment of Pol32 in yeast, human and fly. Pol32 was originally identified in Drosophila melanogaster by Gray et al. (2004) based on its pol-alpha interacting domain (highlighted) and PCNA-interacting motif (underlined). Only the first 14 amino acids would be present in a theoretically expressed pol32L2. (TIF) [file pgen.1002659.s001.tif]

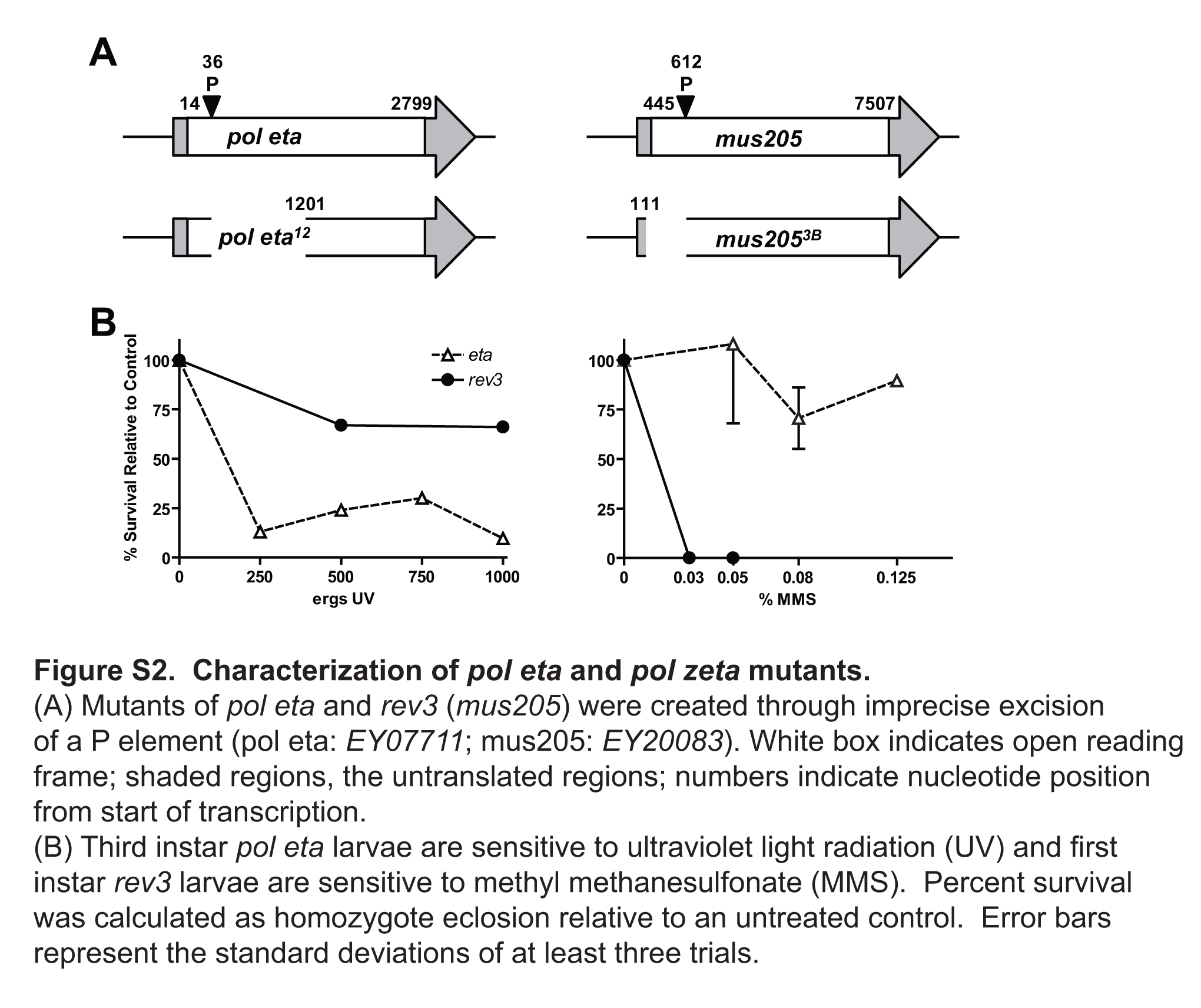

Supplement: Figure S2 — Characterization of pol eta and pol zeta mutants. (A) Mutants of pol eta and rev3 (mus205) were created through imprecise excision of a P element (pol eta: EY07711; mus205: EY20083). White box indicates open reading frame; shaded arrow, the untranslated regions; numbers indicate nucleotide position from start of transcription. (B) pol eta mutants are sensitive to ultraviolet radiation (UV) and rev3 mutants are sensitive to methyl methanesulfonate (MMS). Percent survival was calculated as homozygote eclosion relative to an untreated control. Error bars represent the standard deviations of at least three trials. (TIF) [file pgen.1002659.s002.tif]

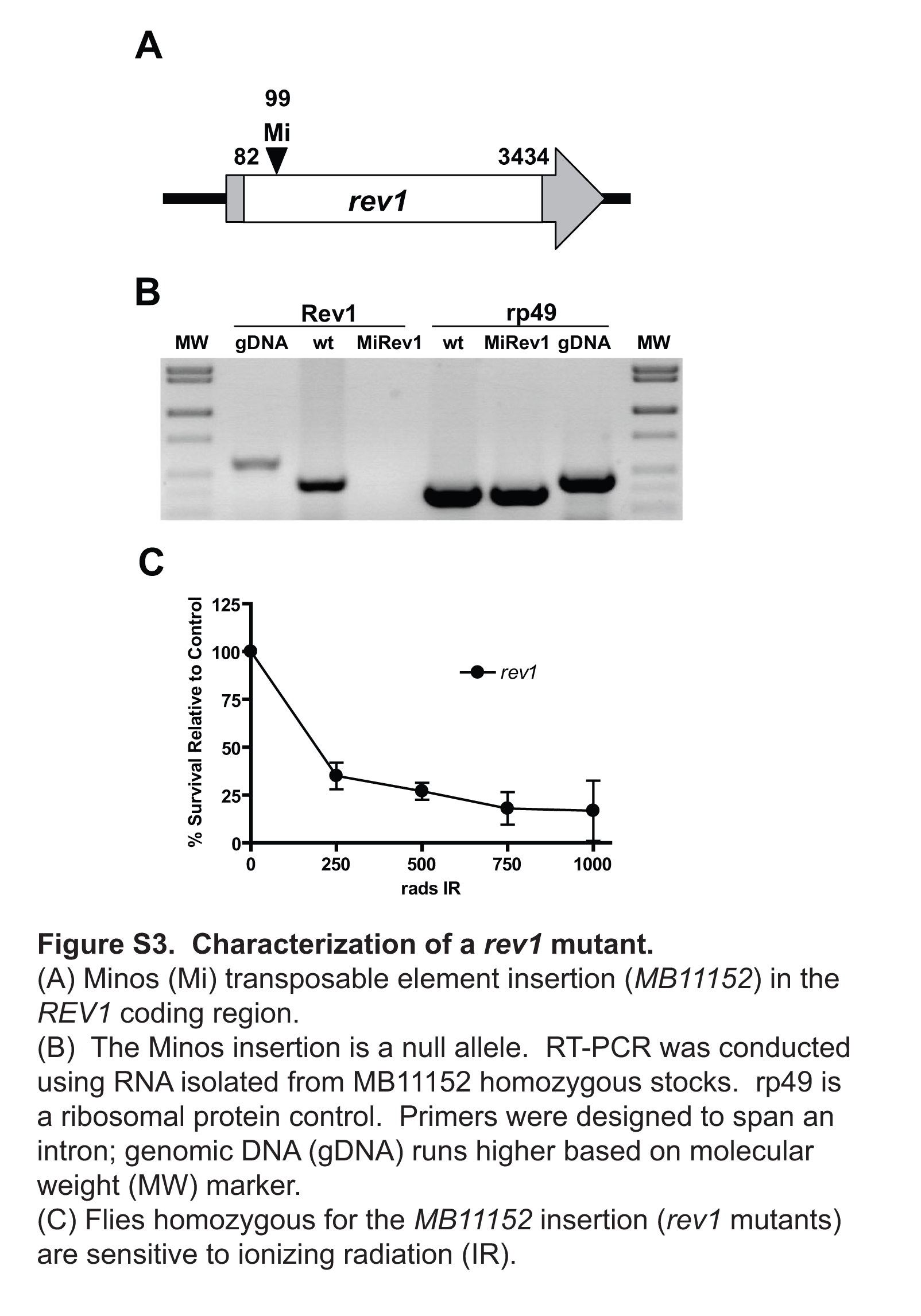

Supplement: Figure S3 — Characterization of a rev1 mutant. (A) Minos (Mi) transposable element insertion (MB11152) in the REV1 coding region. (B) The Minos insertion is a null allele. RT-PCR was conducted using RNA isolated from MB11152 homozygous stocks. rp49 (ribosomal protein) was used as a control. Primers were designed to span an intron; PCR using genomic DNA (gDNA) produces a larger PCR product. MW = molecular weight marker. (C) Flies homozygous for the MB11152 insertion (rev1 mutants) are sensitive to ionizing radiation (IR). (TIF) [file pgen.1002659.s003.tif]

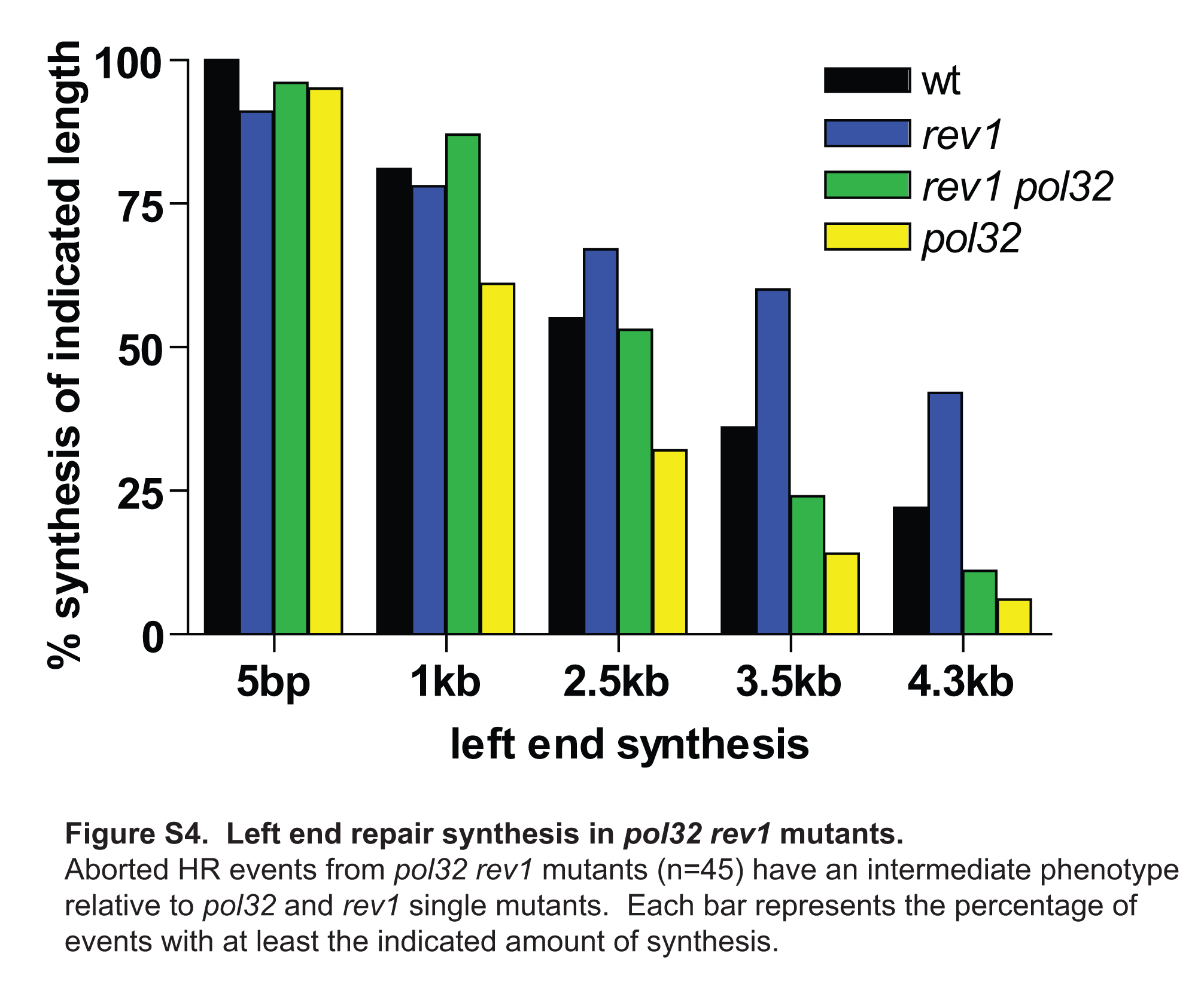

Supplement: Figure S4 — Left end repair synthesis in pol32 rev1 mutants. Aborted HR events from pol32 rev1 mutants (n = 45) have an intermediate phenotype relative to pol32 and rev1 single mutants. Each bar represents the percentage of events with at least the indicated amount of synthesis. (TIF) [file pgen.1002659.s004.tif]
