# Supplementary material for: Competition between Replicative and Translesion Polymerases during Homologous Recombination Repair in Drosophila
Source: PLoS Genet. 2012 Apr 19;8(4):e1002659. doi: 10.1371/journal.pgen.1002659 (PMC3330096; doi:10.1371/journal.pgen.1002659)
Supplement: Text S1 — Additional Methods. Expanded methods, including primers used, for the P{wa} assay. (DOC) [file pgen.1002659.s005.doc]

### **Text S1. Additional Methods.**

***P{w^a^}* assay repair synthesis tract length PCR**

### For product sizes of ~1kb or less (left: 5bp, 300bp, and 1kb; right: 5bp, 250bp, 1kb, 2.5kb, and 4.5kb), 1 μL of fly DNA was used in a 20 μL PCR reaction volume (10 mM Tris-HCl (pH 9.0), 50mM KCL, 2.5mM MgCl2, 0.1% Triton X-100, 1.25 μM each primer, 250 μM dNTPs and *Taq* DNA polymerase). For all other product sizes >1kb (remaining left end distances, right end 3.5kb, 4.3kb), 1.25 μL of fly DNA was used in a 25 μL PCR reaction volume using LongAmp® *Taq* DNA polymerase and buffer (NEB). PCR products were analyzed by 1% agarose gel electrophoresis and ethidium bromide staining.

### Left end synthesis: The forward primer Sd5471 (CCCTGTCTGAACTTCCGTAG) was used with each of the following reverse primers:

### POUT (5bp, CCGCGGCCGCGGACCACCTTATGTTATTTC);

### left317R (300bp, CAGCCTTCCACTGCGAATCA);

### left1038R (1kb, TTGCCGAACTGGAGGTAAGCTC);

### left2561R (2.5kb, GCTCGCATATCTGGCTCTAAGAC);

### left3562R (3.5kb, TCAGACAACGGTGAGTGGTTCC);

### left4327R (4.3kb, TGCCACGACATCTGACCTATCG);

### left4561R (4.6kb, AACTTTTGGCCGTGATGGGCAG).

Right end synthesis:

Primer sets Sd5941a (GCCTTGCTTCTTCCACACAGCGTG) and POUT were used for 5bp. The forward primer Sd5678a (CCCTCGCAGCGTACTATTGAT) was used with reverse primers Pwa248a (250bp, GTCGACCTGCAGCCAAGCTTTG) and Pwa997a (1kb, GATGTTGCAATCGCAGTTC).

The forward primer Pwa1487 (CGTTGTTTGCACGTCTCGCTCG) was used with the following reverse primers:

Pwa2420a (2.5kb, GAGCGAGATGGCCATATGGCTG),

Pwa3451a (3.5kb, GCCACCTCATAAGACAATTGCG) and

Pwa4339a (4.3kb, CTCACCGTTCTTGAGCAAATG).

Primer sets Pwa4287 (GCAACGAGCGACACATACCG) and Pwa4674a (GGACTGGGCCCATAACCTGTTG) were used for 4.6kb.
